# COVID-19-related prescribing challenge in intellectual disability

**DOI:** 10.1192/bjo.2021.26

**Published:** 2021-03-19

**Authors:** Bushra Rauf, Hafsa Sheikh, Hassan Majid, Ashok Roy, Rani Pathania

**Affiliations:** Intellectual Disability Psychiatry, Coventry and Warwickshire Partnership NHS Trust, UK; Intellectual Disability Psychiatry, Coventry and Warwickshire Partnership NHS Trust, UK; Intellectual Disability Psychiatry, Coventry and Warwickshire Partnership NHS Trust, UK; Intellectual Disability Psychiatry, Coventry and Warwickshire Partnership NHS Trust, UK; Intellectual Disability Psychiatry, Coventry and Warwickshire Partnership NHS Trust, UK

**Keywords:** Intellectual disability, COVID-19, psychotropic medications, lockdown, autism spectrum disorders

## Abstract

**Background:**

The COVID-19 pandemic and associated restrictions are expected to affect the mental health of the population, especially people with intellectual disability and/or autism spectrum disorder, because of a variety of biological and psychosocial reasons.

**Aims:**

This study aimed to estimate if COVID-19 restrictions are associated with a change in number of total consultations carried out by psychiatrists and prescription of psychotropic medication in people with intellectual disability and/or autism spectrum disorder, within a community intellectual disability service.

**Method:**

A quantitative observational study was conducted, involving retrospective and prospective data collection before and during lockdown. Data was collected on a spreadsheet and emailed to all psychiatrists working within the Coventry and Warwickshire Partnership NHS Trust-wide community intellectual disability service. Variables included total consultations, medication interventions, types of medications used, multidisciplinary team input and clinical reasons for medication interventions. Data was analysed separately for child and adolescent mental health services (CAMHS) and adult intellectual disability teams, and for the whole service.

**Results:**

During the lockdown period, total consultations in the community intellectual disability service increased by 19 per week and medication interventions increased by two per week. Multidisciplinary team input increased in CAMHS from 0.17 to 0.71 per week and in adult intellectual disability from 5.7 to 6.5 per week. Hypnotics and benzodiazepines were the most commonly prescribed psychotropic medications during the lockdown period.

**Conclusions:**

COVID-19-related lockdown resulted in an increase in medication interventions, total consultations and involvement of multidisciplinary teams to manage mental health and behavioural issues in people with intellectual disability and/or autism spectrum disorder.

Intellectual disability is a condition characterised by significant impairments of both intellectual and adaptive functioning and an onset before 18 years of age.^1^ The UK Government uses the term ‘learning disability’ for this condition. Intellectual disability affects almost 1–2% of the general population.^2^ The degree of intellectual disability is classified as mild, moderate, severe or profound, with over 90% of those affected falling within the mild range.^3^ People with an intellectual disability have a high rate of mental health comorbidity, with a point prevalence of around 30%.^4^ They develop psychiatric conditions at rates similar to or higher than the general population.^5^ They also have high rates of physical health comorbidity and premature mortality.^6,7^ A significant proportion of people with intellectual disability display ‘behaviours that challenge’, defined as ‘behaviours of an intensity, frequency, or duration that threaten the physical safety of the person or others or restrict access to community facilities’.^8^ Psychotropic medication is widely used to treat mental and behavioural disorders in people with intellectual disability. There are concerns that these drugs, in particular antipsychotics, are being used inappropriately in people with intellectual disability for the treatment of behaviours that challenge.^9–12^ These concerns were amplified by the Department of Health inquiry into Winterbourne View Hospital, which highlighted the inappropriate use of psychotropic medication.^13^ Inappropriate medication use in in people with intellectual disabilities was highlighted by the National Health Service (NHS) England^14^ as an area for development, under the Transforming Care Programme in 2015. The Royal College of Psychiatrists fully supported the STOMP (Stopping Overmedication of People with a Learning Disability, Autism or Both) campaign by NHS England in 2016,^15^ and pledged to work with its partners to promote the campaign in leading to a reduction in the use of psychotropic medication in people with intellectual disability.^16^ It included the prescription of any psychotropic medication, including antipsychotics, antidepressants, anxiolytics and mood stabilisers, setting out a framework for clinicians on how to rationalise prescribing and, where appropriate, taper and stop psychotropic medications.^16^

## Aims

Psychotropic medication reduction has been achieved in number of patients seen in general and specially created behavioural clinics established throughout the service triggered by the national STOMP programme. However, the COVID-19 pandemic in 2020 has changed the environment and social circumstances of patients with intellectual disability and/or autism spectrum disorder, with consequent potential impact on their mental health and behaviours. It was anticipated that lockdown restrictions, such as closure of day centres, inability to access community for routine activities, and other factors like carer/staff time off owing to health/issues with shielding, were likely to have a negative effect on patient mental well-being, leading to stress and worsening of mental health and behaviours that challenge. COVID-19 restrictions have also limited the form in which local mental health services are delivered, making provision of face-to-face psychological interventions more difficult. Consequently, it was anticipated that the use of psychotropic medication could increase during this time, to manage behavioural crisis, prevent placement breakdown and maintain the safety of people with intellectual disability and/or autism spectrum disorder, carers and families.

The study aimed to determine the impact of COVID-19 lockdown restrictions on overall total consultations and use of psychotropic medications in people with intellectual disability and/or autism spectrum disorder, in the service which served a population of approximately 1.1 million people through a combination of adult and child and adolescent mental health services (CAMHS) community intellectual disability teams.

## Method

Following an increase in COVID-19 infection rate, lockdown restrictions were imposed in the UK on 23 March 2020.^17^ Many restrictions were eased after the release of updated government guidance in July 2020.^18^

An observational quantitative study was carried out, involving data collection over a 6-month period, including a 12-week pre-lockdown (1 January to 22 March 2020) and 14-week lockdown period (23 March to 30 June 2020). Data was collected by emailing a Microsoft Excel spreadsheet (office 365 for Windows) to all psychiatrists in community CAMHS and adult intellectual disability services in the Coventry and Warwickshire Partnership NHS Trust. Clinicians were asked to provide information for each patient reviewed during working hours for the pre-lockdown and lockdown period. This included date reviewed; medications used and if they increased, commenced or changed; summary of clinical decision, including the main reason for medication alteration (if planned beforehand or related to COVID-19); and other multidisciplinary team (MDT) involvement (planned or COVID-19-related). Clinicians were also requested to provide total numbers of psychiatry consultations carried out during the pre-lockdown and lockdown periods. This survey was deemed to be a service evaluation, and so ethics approval was not sought. This study was an observational study/survey based on patients data with no direct patient involvement/intervention hence patients consent was not required.

Data were analysed by comparing the pre-lockdown with the lockdown period, using the following variables: total number of consultations for both children and adults, total numbers of medication interventions for both children and adults, and total number of contacts of MDT input (planned/unplanned) for both children and adults.

## Results

During the pre-lockdown period there were 1218 psychiatric consultations across the service (133 in CAMHS and 1085 in adult services), amounting to an average of 103 consultations per week. During the lockdown period there were 1691 psychiatric consultations across the service (227 in CAMHS and 1464 in adult services), amounting to an average of 118 consultations per week, an increase of 14.5%.

Medication interventions took place 163 times out of 1218 pre-lockdown consultations (13 per week), and 211 times out of 1691 lockdown consultations (15 per week).

### CAMHS intellectual disability community service results

There were 360 CAMHS intellectual disability consultations during the whole period of 6 months, with 133 occurring during the 12-week pre-lockdown period (11 per week) and 227 during the 14-week lockdown period (16 per week).

Out of the 121 medication interventions, 48 (average of 4 per week) occurred pre-lockdown and 73 (average of 5 per week) occurred during lockdown. Medication interventions as a ratio of total consultations remained almost the same during pre-lockdown and lockdown, at 0.32 per week (48/133) and 0.32 per week (73/227), respectively. Overall medication intervention types within CAMHS that were recorded as increased, commenced and changed almost remained the same in both periods (Table 1).

**Table 1:** Medication intervention type

	Increased	Commenced	Changed	Total
CAMHS intellectual disability			
Pre-lockdown	22 (46%)	18 (38%)	8 (17%)	48
Lockdown	33 (45%)	27 (37%)	13 (18%)	73
Total				121
Adult intellectual disability			
Pre-lockdown	82 (69%)	33 (28%)	3 (3%)	118
Lockdown	74 (51%)	60 (42%)	10 (7%)	144
Total				262
CWPT intellectual disability			
Pre-lockdown	104 (64%)	51 (31%)	8(5%)	163
Lockdown	105(50%)	87(41%)	19 (9%)	211
Total				374

CAMHS, child and adolescent mental health services; CWPT, Coventry and Warwickshire Partnership NHS Trust.

During both pre-lockdown and lockdown, antipsychotics were the most commonly prescribed medication, followed by hypnotics/benzodiazepines and then antidepressants/attention-deficit hyperactivity disorder (ADHD) medications (Table 2). Hypnotics/benzodiazepines were the most commonly prescribed psychotropic medications for COVID-19-related issues. However, ADHD, antipsychotics, antidepressants, mood stabilisers and other medications were altered or increased as part of a longer-term plan (Table 3).

**Table 2:** Medication type

Types of medications	Pre-lockdown	Lockdown
CAMHS intellectual disability		
Antipsychotics	64%	57%
Hypnotics/benzodiazepines	22%	39%
Antidepressants	18%	12%
ADHD	16%	15%
Mood stabilisers	7%	1%
Others (EPSE, antiepileptics)	7%	1%
Adult intellectual disability		
Antipsychotics	32%	27%
Hypnotics/benzodiazepines	31%	50%
Antidepressants	33%	24%
ADHD	7%	2%
Mood stabilisers	4%	4%
Others (EPSE, dementia, antiepileptic)	1%	3%

CAMHS, child and adolescent mental health services; ADHD, attention-deficit hyperactivity disorder; EPSE, extra pyramidal side effects.

**Table 3:** Rationale for different types of medication used during lockdown period

	Pre-lockdown	Lockdown	Non-COVID-19/planned	COVID-19 response
CAMHS intellectual disability				
Antipsychotics	29	38	23 (61%)	15 (39%)
Antidepressants	8	8	5 (62%)	3 (38%)
Mood stabilisers	3	1	1 (100%)	0 (0)%
Hypnotics/benzodiazepines	10	26	8 (31%)	18 (69%)
ADHD	7	10	9 (90%)	1 (10%)
Others (epilepsy, EPSE)	3	1	1 (100%)	0 (0%)
Adult intellectual disability				
Antipsychotics	38	39	4 (10%)	35 (90%)
Antidepressants	39	35	5 (14%)	30 (86%)
Mood stabilisers	5	6	0 (0%)	6 (100%)
Hypnotics/benzodiazepines	37	72	9 (12%)	63 (88%)
ADHD	7	2	2 (100%)	0 (0%)
Others (epilepsy, EPSE, dementia)	2	7	7 (100%)	0 (0%)

CAMHS, child and adolescent mental health services; ADHD, attention-deficit hyperactivity disorder; EPSE, extra pyramidal side effects.

Documented reasons for medication changes to manage challenging behaviour and/or mental health issues during the lockdown period were mostly triggered by a change in routine, staying indoors, cancelled holidays or worsening sleep patterns. However, in some cases the overall clinical presentation during lockdown was improved, possibly because of reduced demands on children and adolescents, resulting in some medication, like those for ADHD and sleep, being either reduced or stopped.

Before lockdown, MDT input was in 2 contacts out of 133 consultations (0.17 per week). During lockdown, MDT input was in 10 contacts out of 227 consultations (0.71 per week). This fourfold-per-week increase in MDT input predominantly involved community nursing, occupational therapy and psychologists (Table 4). However, in data from some teams, information about the full range of MDT disciplines was not available.

**Table 4:** Multidisciplinary team disciplines

CWPT intellectual disability	Community nursing	Psychologist	Occupational therapist	Speech and language therapist	Intensive support team/care and treatment reviews	Physiotherapist	Social care	Support workers
CAMHS intellectual disability								
Pre-lockdown	1 (2%)	1 (2%)	1 (2%)	0	0	0	0	0
Lockdown	8 (12%)	3 (4%)	8 (12%)	3 (4%)	0	0	0	0
Adult intellectual disability								
Pre-lockdown	25 (21%)	17 (14%)	8 (7%)	8 (7%)	4 (3%)	1 (0.8%)	3 (3%)	2 (2%)
Lockdown	41 (28%)	23 (16%)	10 (7%)	4 (3%)	8 (6%)	1 (0.7%)	0	4 (3%)

CWPT, Coventry and Warwickshire Partnership NHS Trust; CAMHS, child and adolescent mental health services.

### Adult intellectual disability community service results

There are five adult intellectual disability community teams within the Trust catchment area, covering a population of 1.1 million.

There were 2549 consultations during the whole 6-month period, with 1085 (90 consultations per week) occurring pre-lockdown and 1464 (105 consultations per week) occurring during lockdown. Out of 1085 pre-lockdown consultations, there were 118 medication interventions (9 changes per week), and out of 1464 lockdown consultations, there were 144 medication interventions (10 changes per week). Medication interventions as a ratio of total consultations remained almost the same during both pre lockdown and lockdown, at 0.11 per week (118/1085) and 0.1 per week (144/1464), respectively. Overall medication interventions within adult intellectual disability services that were recorded as commenced, increased and changed showed that the majority of medications were increased in pre-lockdown (Table 1), whereas the majority of medications were commenced and changed in lockdown.

Analysis of the clinical reason for using medications during the lockdown period showed that hypnotics/ benzodiazepines were the most commonly prescribed psychotropic medications for COVID-19-related issues, followed by antipsychotics, antidepressants and mood stabilisers (Tables 2 and 3). However, ADHD and other medications were altered as part of a longer-term plan (Table 3).

Documented reasons for medication changes during the lockdown period were mostly issues related to low mood, anxiety, psychosis, self-harm, aggression and poor sleep, likely a result of changes in routine, restrictions forcing people to stay at home, limited community access, inability to meet family, shielding of patient or parents, and closure of day centres and colleges.

MDT input went up from 68 contacts out of 1085 consultations in pre-lockdown (5.7 per week) to 91 contacts out of 1464 in the lockdown period (6.5 per week). This small increase in MDT input predominantly involved community nursing, occupational therapy, psychologists and the intensive support team (Table 4). However, data about the full range of MDT disciplines were not available from some teams.

## Discussion

Overprescription of psychotropic medication without diagnostic justification was the main driver for the launch of the STOMP campaign. During the pandemic, patients continued to have routine psychiatric follow-up via telephone, face-to-face and video consultations. Psychiatric consultations increased by 14.5% per week in the lockdown period because of an increase in urgent assessments, as routine appointments remained the same during both periods. The reasons for urgent psychiatric assessments were worsening of mental health and behaviour of patients with intellectual disability with or without autism spectrum disorder that was attributed by carers to changes in their environment and daily routine, secondary to restrictions owing to lockdown. Other factors influencing the increase in psychiatry consultations and medication prescription included limited availability of service provision because of lockdown restrictions that resulted in reduction in face-to-face contacts from other professionals (including therapy groups conducted by psychologists and nurses), and redeployment of community professionals to in-patient services.

Pharmacological interventions were increased throughout the intellectual disability service by two per week during lockdown. Sleep disturbance and increased anxiety resulted in increased commencement and use of higher doses of hypnotics and benzodiazepines. The nature and severity of urgent consultations requiring medication intervention were mainly to prevent placement breakdown and hospital admissions, and to maintain the safety of patients and/or carers. However, in some CAMHS patients with intellectual disability, overall clinical presentation was improved, possibly because of reduced demands and expectations during the lockdown period. Hence for children, medication for ADHD and sleep (such as melatonin) was either reduced or stopped. During pre-lockdown, worsening of mental health and/or behaviour was observed in some, possibly because of anticipatory anxiety related to COVID-19, the expected lockdown and its effect on day-to-day life. This increase in the number of medications being commenced or increased during both periods reflects a possible reversing the gains achieved after the launch of the STOMP initiative.

MDT input was increased by almost one per week for adult intellectual disability services and fourfold per week for CAMHS intellectual disability services during lockdown, albeit from a low baseline. Although community nursing accounted for most of the extra input, followed by psychology and then occupational therapy, these data may not be an accurate reflection because some teams were unable to provide the details of input by individual discipline. Increased demand for community nursing support may reflect an increased in need to review changes in mental health and behaviour, and monitoring response to and side-effects of medication changes. Intensified psychology input could be an expected response to increased demand for behavioural support and anxiety management related to COVID-19 restrictions. Increased occupational therapy input could be linked to advising patients and carers how to develop or access appropriate in-house and online daily activities during lockdown. During the lockdown period, in some community teams, there was also an increase in number of intensive support team contacts and care and treatment review requests, to prevent possible hospital admissions or to hasten discharge.

### Strengths and limitations

There are three key methodological limitations in this study. First, the duration of the pre-lockdown period (12 weeks) is less than the lockdown period (14 weeks), which tends to inflate the figures for the second time period. This is compensated for by presenting data as a weekly rate. Second, complete data for MDT intensification was not available from all teams, especially with regards to the input of different professionals in the MDT. Third, in this study the same patients were not compared in pre-lockdown and lockdown periods. However, this could be considered in any future research projects carried out during future expected COVID-19 waves. This would allow for the application of appropriate statistical tests for data analysis from the outset.

It was not possible to clearly ascertain if medication change was directly attributable to the pandemic and its restrictions or to other independent factors, such as deterioration of mental or physical health and side-effects owing to current medication.

There are several strengths to our study, namely that this is a unique study that was designed and carried out in a short period of time, relevant to the exceptional and urgent circumstances related to the COVID-19 pandemic. Also, this study was also carried out throughout a large and diverse specialist intellectual disability service, with a good sample size.

### Recommendations

We make several recommendations based on the results of this study. First, the reduction of overprescribing of antipsychotic medication in people with intellectual disability achieved after the launch of the STOMP campaign should not be lost during the pandemic. Clinicians should continue to conduct regular multidisciplinary reviews to support reduction and possible withdrawal of medication that has been commenced or increased during the COVID-19 period without clear agreed diagnostic justification. The development of a shared formulation and a behaviour support plan could avoid excessive reliance on medication. There needs to be improved monitoring of potential short- and long-term neurologic and metabolic side-effects from psychotropic medications.
Second, in view of continuing restrictions during the pandemic, intellectual disability services should be prepared to implement effective remote video consultation for routine appointments and provide adequate personal protective equipment to enable better assessments, observations and treatments.
Third, maintaining adequate numbers of community psychiatrists, supported by appropriate MDTs, should be a priority to meet the expected increase in consultations and requests for medication interventions to prevent placement breakdowns and hospital admissions, and to maintain the safety of patients and carers. The wider implications of redeploying skilled community intellectual disability staff needs to be carefully considered as it could reduce availability of appropriate psychosocial interventions in preference to medication.
Finally, the study should be replicated with the same sample being monitored over a longer period of time through the ongoing pandemic and beyond, to look at factors that could lead to unnecessary use of psychotropic medication in preference to psychological, behavioural and environmental measures to deal with distress experienced by people with intellectual disability living through the pandemic. This could enable identification of patient, carer and environmental factors that influence the nature of support and treatment provided.

## Data Availability

The data that support the findings of this study are available from the corresponding author, B.R., upon request.

## References

[1] World Health Organization (WHO). International Statistical Classification of Diseases and Related Health Problems (ICD-10). WHO, 2008.

[2] Emerson E, Hatton C, Robertson J, Roberts H, Baines S, Evison F, People with Learning Disabilities in England 2011: Services and Support. Improving Health and Lives Learning Disabilities Observatory, 2011 (https://www.glh.org.uk/pdfs/PWLDAR2011.pdf).

[3] Department of Health. Valuing People: A New Strategy for Learning Disability for the 21st Century. Department of Health, 2001. https://www.gov.uk/government/publications/valuing-people-a-new-strategy-for-learning-disability-for-the-21st-century

[4] Cooper S-A, Smiley E, Morrison J, Williamson A, Allan L. Mental ill-health in adults with intellectual disabilities: prevalence and associated factors. Br J Psychiatry 2007; 190: 27–35. 17197653 10.1192/bjp.bp.106.022483

[5] Buckles J, Luckasson R, Keefe E. A systematic review of the prevalence of psychiatric disorders in adults with intellectual disability, 2003–2010. J Ment Health Res Intellect Disabil 2013; 6: 1181–207.

[6] Heslop P, Blair P, Fleming P, Hoghton M, Marriott A, Russ L, Confidential Inquiry into Premature Deaths of People with Learning Disabilities (CIPOLD). Lancet 2013; 383(9920): 889–95. 24332307 10.1016/S0140-6736(13)62026-7

[7] Public Health England. People with Learning Disabilities in England 2015: Main Report. Public Health England, 2016 (https://assets.publishing.service.gov.uk/government/uploads/system/uploads/attachment_data/file/613182/PWLDIE_2015_main_report_NB090517.pdf).

[8] Emerson E, Kiernan C, Alborz A, Reeves D, Mason H, Swarbrick R, The prevalence of challenging behaviours: a total population study. Res Dev Disabil 2001; 22: 77–93. 11263632 10.1016/s0891-4222(00)00061-5

[9] Molyneux B, Emerson E, Caine A. Prescription of psychotropic medication to people with intellectual disabilities in primary healthcare settings. J Appl Res Intellect Disabil 1999; 12: 46–57.

[10] Matson JL, Bamburg JW, Mayville EA, Pinkston J, Bielecki J, Kuhn D, Psychopharmacology and mental retardation – a 10 year review (1990–1999). Res Dev Disabil 2000; 21: 263–96. 10983783 10.1016/s0891-4222(00)00042-1

[11] Brylewski J, Duggan L. Antipsychotic medication for challenging behaviour in people with learning disability. Cochrane Database Syst Rev 2004; 3: CD000377. 10.1002/14651858.CD000377.pub2 PMC12334078 15266428

[12] Tsiouris JA. Pharmacotherapy for aggressive behaviours in persons with intellectual disabilities – treatment or mistreatment? J Intellect Disabil Res 2010; 54: 1–16. 10.1111/j.1365-2788.2009.01232.x 20122096

[13] Department of Health. Transforming Care: A National Response to Winterbourne View Hospital: Department of Health Review Final Report. Department of Health, 2012 (https://assets.publishing.service.gov.uk/government/uploads/system/uploads/attachment_data/file/213215/final-report.pdf).

[14] NHS England. Urgent Action Pledged on Over-Medication of People with Learning Disabilities. NHS England, 2015 (http://www.england.nhs.uk/2015/07/14/urgent-pledge).

[15] NHS England. Stopping Overmedication of People with a Learning Disability, Autism or Both. NHS England, 2016 (https://www.england.nhs.uk/learning-disabilities/improving-health/stomp/).

[16] Alexander R, Devapriam J, Branford D, Roy A, Sheehan R, Anand E, *Psychotropic Drug Prescribing for People with Intellectual Disability, Mental Health Problems and/or Behaviours that Challenge: Practice Guidelines* (2016). Faculty Report FR/ID/09, Royal College of Psychiatrists, 2016 (http://www.rcpsych.ac.uk/pdf/FR_ID_09_for_website.pdf).

[17] UK Government. *Staying at Home and Away from Others (Social Distancing)*. UK Government, 2020 (https://www.gov.uk/government/publications/full-guidance-on-staying-at-home-and-away-from-others).

[18] UK Government. *Coronavirus (COVID-19): Guidance & Support. 1 July 2020*. UK Government, 2020 (https://www.gov.uk/guidance/national-lockdown-stay-at-home).

